# Physicians’ perspectives on clinical pharmacy services in Northern Sweden: a qualitative study

**DOI:** 10.1186/s12913-018-2841-3

**Published:** 2018-01-24

**Authors:** Charlotta Vinterflod, Maria Gustafsson, Sofia Mattsson, Gisselle Gallego

**Affiliations:** 1 Department of Pharmacology and Clinical Neuroscience, Umeå University, SE-90187 Umeå, Sweden; 2 School of Medicine, The University of Notre Dame Australia, 160 Oxford Street, Darlinghurst, NSW 2010 Australia

**Keywords:** Clinical pharmacy, Physicians, Collaboration, Hospital, Sweden

## Abstract

**Background:**

In many countries, clinical pharmacists are part of health care teams that work to optimize drug therapy and ensure patient safety. However, in Sweden, clinical pharmacy services (CPSs) in hospital settings have not been widely implemented and regional differences exist in the uptake of these services. Physicians’ attitudes toward CPSs and collaborating with clinical pharmacists may facilitate or hinder the implementation and expansion of the CPSs and the role of the clinical pharmacist in hospital wards. The aim of this study was to explore physicians’ perceptions regarding CPSs performed at hospital wards in Northern Sweden.

**Methods:**

Face-to-face semi-structured interviews were conducted with a purposive sample of nine physicians who had previously worked with clinical pharmacists between November 2014 and January 2015. Interviews were digitally recorded, transcribed and analysed using a constant comparison method.

**Results:**

Different themes emerged regarding physicians’ views of clinical pharmacy; two main interlinked themes were service factors and pharmacist factors. The service was valued and described in a positive way by all physicians. It was seen as an opportunity for them to learn more about pharmacological treatment and also an opportunity to discuss patient medication treatment in detail. Physicians considered that CPSs could improve patient outcomes and they valued continuity and the ability to build a trusting relationship with the pharmacists over time. However, there was a lack of awareness of the CPSs. All physicians knew that one of the pharmacist’s roles is to conduct medication reviews, but most of them were only able to describe a few elements of what this service encompasses. Pharmacists were described as “drug experts” and their recommendations were perceived as clinically relevant. Physicians wanted CPSs to continue and to be implemented in other wards.

**Conclusions:**

All physicians were positive regarding CPSs and were satisfied with the collaboration with the clinical pharmacists. These findings are important for further implementation and expansion of CPSs, particularly in Northern Sweden.

**Electronic supplementary material:**

The online version of this article (10.1186/s12913-018-2841-3) contains supplementary material, which is available to authorized users.

## Background

Problems associated with drug treatment such as adverse drug reactions (ADRs), medication errors and adverse drug events (ADEs) are common. ADEs can result in drug-related morbidity and mortality, and studies show that from seven up to approximately 30% of hospital admissions are directly related to drug treatment problems [1–5]. According to a study conducted in Northern Sweden in 2016, as much as 41% of hospital admissions were judged as drug-related among old people with dementia [6]. Moreover, according to one meta-analysis, up to 24% of patients develop ADRs during their hospital stay [7].

There is a clear need for interventions that decrease drug-related problems (DRPs) and improve patient safety, for example by implementing clinical pharmacy services (CPSs) in hospitals. A systematic review conducted by Graabaek et al., described that clinical pharmacy services in the hospital ward resulted in positive effects on medication use, health service use and costs [8]. Furthermore, research has shown that collaboration between physicians and pharmacists has the potential to reduce DRPs, emergency department visits and hospitalization [8–14]. For example a study by Gillespie et al., demonstrated that including a clinical pharmacist in a health care team reduced drug-related readmissions by 80% among people 80 years and older in an internal medicine ward in Uppsala, Sweden [15].

Although clinical pharmacists have been an integral part of health care for several years in countries like the United States [9], CPSs have not been widely implemented across Swedish hospitals. This applies particularly to Northern Sweden. Västerbotten County is one of five counties situated in Norrland, the most northerly region of Sweden. Norrland is the largest geographical area, covering about 59% of Sweden’s total surface area. The population living in the region is about 12% of Sweden’s total population of approximately 10 million [16, 17]. The relatively small numbers of patients dispersed over large geographic areas, together with shortage of health care professionals pose special challenges for the provision of health services in this region. Each of Sweden’s 21 county councils sets their own strategies and policies for the inclusion of clinical pharmacy. Most of the county councils that employ clinical pharmacists are based in Southern Sweden and therefore only certain cities and hospitals have access to CPSs around Sweden.

Physicians’ attitudes toward CPSs and collaborating with clinical pharmacists may facilitate or hinder the implementation and expansion of the CPSs and the role of the clinical pharmacist in hospital wards. To date, however, there have been no qualitative studies exploring physicians’ perceptions of CPSs in hospital settings in Sweden. The aim of this study was therefore to explore physicians’ perceptions regarding CPSs performed in hospitals operated by Västerbotten County Council.

## Method

This study took place in three hospital wards (one geriatric ward and two internal medical wards) in Norrlands University Hospital, situated in Västerbotten County, Sweden.

### Context

CPS started as a project within Västerbotten County Council in 2002. Today, there are six clinical pharmacists active in inpatient and outpatient care in Västerbotten. The service provided by the clinical pharmacists working in inpatient care consists of medication reconciliation, medication review (sometimes including patient counselling and participation in ward rounds. Table 1 describes the CPSs.

**Table 1:** Clinical pharmacy services provided in Northern Swedish hospitals

When	How often	Activity
At admission	Once for each patient	Admission medication reconciliation
During hospital stay	As long as the patient is still in the hospital ward	Medication review and monitoring
	In some wards, depending on need and time	Patient counselling
	When clinical relevant DRPs are found	Discussion with responsibly physician (for example participating in ward rounds)
At discharge	Occasionally	Discharge medication reconciliation

### The interview guide

An interview guide was developed based on a review of the literature and discussions within the research team. The schedule focused on three main topics: the pharmacist role on the ward, clinical outcomes of CPSs, and future role of CPS (See Additional file 1). A pilot test interview was conducted to determine the suitability of the questions. After the test, interview questions that the respondent thought were difficult to answer were changed or reworded. The pilot interview also served as a learning process for the interviewer and gave the opportunity to improve the interviewing schedule.

### Participant selection

A purposive sample, that is, a sample that would serve the purpose of the study was used [18]. Physicians who had previously worked with clinical pharmacists, on five different wards, and who were familiar with the type of services pharmacists provide (i.e. had to have several encounters and interactions with the clinical pharmacists) were invited to participate after approval was granted by the ward managers. Those who did not respond to the first email were sent two reminders. Since data saturation [19] (i.e. no new categories or themes emerged from the data) was achieved after the ninth interview, no further attempts were made to contact those who did not respond.

### Data collection

Face-to-face semi-structured interviews were conducted in Swedish by the first author (CV) between November 2014 and January 2015. The interviews lasted between 15 and 35 min and were based around the interview schedule.

### Data analysis

Interviews were digitally recorded and transcribed. Participants were given the opportunity to read and comment on their transcribed interviews. Only one participant read the transcript but provided no comments. The data analysis was completed in several stages: after each interview, a preliminary data analysis was performed to allow the identification of issues that needed to be further investigated in the following interviews. After this first step, a continuous analysis of collected data was performed and a coding framework was developed by CV and GG. Segments (paragraphs, sentences) were coded and labelled using colours and comments in Microsoft Office Word. Coded segments were then compared for differences and similarities of events and ideas. This process was repeated until all comments were assigned to categories [20].

To verify the identification of themes, the researchers discussed the mapping and coding framework on several occasions. One interview was coded by a second coder (SM). This allowed a clearer definition of categories and further discussion of the meaning of the codes [21]. The coded interviews were compared, differences were discussed by the researchers and a joint decision on what coding should be used for the segment was taken.

## Results

Twenty-two physicians were invited to take part in the study and nine agreed to participate. Two physicians did not have time and no response was received from eleven. As previously mentioned, data saturation was reached after nine interviews so no attempts were made to contact these physicians. The characteristics of the participants are described in Table 2. Most physicians worked in internal medical wards, and a few physicians in a geriatric ward. On average physicians had been working with clinical pharmacists for 4.8 years (range 1–10 years).

**Table 2:** Participant characteristics

Interviewee number	Age	Gender	Years working as physician	Years working with pharmacist	Number of meetings with pharmacist
P1	30–40	Male	4.5	1	10–25
P2	40–50	Female	19	1	10–25
P3	30–40	Female	5	3	10–25
P4	30–40	Male	12	5	> 25
P5	50+	Male	20	1,5	10–25
P6	40–50	Female	22	10	> 10
P7	50+	Male	19	10	> 25
P8	30–40	Female	8	2	10–25
P9	30–40	Female	7	6	10–25

**Fig. 1:**
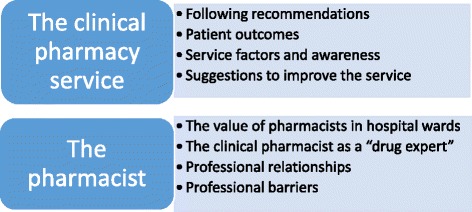
Overview of the main themes that emerged from the qualitative analysis

The interviews drew out a broad range of views regarding CPSs in hospital wards (Fig. 1). To illustrate the findings quotes were selected and translated from Swedish to English. The translations were back-translated by an experienced qualitative researcher who is also fluent in Swedish and English.

### The clinical pharmacy service

All physicians used positive terms to describe the clinical pharmacy service. Some highlighted the fact that they especially appreciated having had the opportunity to discuss each patient systematically with the pharmacist. However, physicians’ views varied; some mentioned that medication review is an important task they would like to perform themselves. But as it saves them time, they are happy to leave that task to the pharmacist. As one of the physicians noted: *“It's a pretty big task to do it [medication review] for every patient, and you might wish that you had the time to do it yourself, but you just can’t cope with that, can you?” (P7).*

A general view voiced by physicians in this study is that medication reviews are time consuming. Some mentioned that medication reviews are not given a high priority, as physicians have to maximize their time and therefore focus mostly on the acute medical problem. As one physician said, *“You can't spend a whole day trying to figure out and read up on and find articles [about interactions] to help one patient.” (P6).*

However, others stated that medication reviews not only “*save them time*” but when also done by a professional perceived to have “*more knowledge of drugs*”. This can be seen in the account from this physician: *“It takes up a good-sized space and saves me a lot of time. Actually, that somebody who knows, and has experience, has gone through it in a structured way, gone through the medication lists.” (P4).*

Another physician commented: *“Then I think it has been very nice, because it has been a forum for discussion. Nobody has been pulling rank, instead it has been an opportunity to talk a little about the patient.” (P5).*

Several of the physicians mentioned that medication review is an opportunity to learn more about drugs and medication treatment. Some of the participants commented that it has helped them become *“better physicians*”.

*“I believe that the work this pharmacist has done has been really good because it has become a revision and a learning process for us about, yes, interactions. It could be specific drugs, how they work together with other drugs or in combination with reduced renal function and things like that, and there is always a need to learn more about this.” (P2)*

#### Following recommendations

Regarding the question of what kind of recommendations the pharmacist provides physicians with, the answers included: drug interactions, dose adjustments, side-effects, inappropriate drugs to certain patient groups (e.g. the elderly), drug changes, combinations of drugs, discontinuation of a drug, administration time, contra-indications, dose reduction and switching to drugs that are better suited based on the patient’s kidney and liver function. The recommendation that was most commonly mentioned was drug interactions. All physicians stated that the recommendations were clinically relevant, adequate and followed most of the time.

*“Usually we say, ‘OK, good let’s do that’, and then we change according to [the pharmacist’s] recommendation. That is the most common, as [the pharmacist] has such sound advice. Sometimes there has been a discussion about a very important drug that has been prescribed and so on, and then we discuss whether there are other options to consider.” (P3)*

Physicians provided different reasons as to why a recommendation was not followed. For example, some mentioned that they had already consulted another specialist, while others said they had already thought about the suggestion before the pharmacist mentioned it and had already decided not to do anything about it. Some of the younger physicians noted that they follow the recommendations made by other specialist or a more experienced physician rather than the one provided by the pharmacist.

#### Patient outcomes

All physicians perceived medication reviews performed by a pharmacist to have a positive impact on patient outcomes. Some mentioned that while some benefits could be obtained while the patient is in the hospital, the impact of medication reviews once the patient is discharged from hospital cannot be determined. Patient outcomes attributed to the clinical pharmacy service included: reduced risk of side-effects and interactions, and reduced length of stay. Both physicians who have been practising medicine for a long time and those who have been practising for a shorter time shared this view. One physician also described the clinical pharmacy service as an opportunity to perform a “*safety check*” on the patients’ medication treatment.

“*The patient safety you can observe (…) is reduced side-effects, reduced risk of interactions and so forth*.” “*The better the medications are balanced for the patient, the better it is. (…) I believe it is a patient-safety issue.*” *(P4)*

#### Service factors and awareness

When participants were asked what they thought the pharmacist does on the ward, they gave different answers. Some specified many different tasks while others did not. There were differences based on the experience and years working with the pharmacists. Many of the older physicians were able to describe the tasks performed by the pharmacists before the ward round. However, most of the younger physicians described what happens during the actual ward rounds. One of the younger participants mentioned *“I don’t really know what she [the pharmacist] does.” (P3).*

Tasks that were described included: the pharmacists look at patients’ medical records, share drug knowledge, establish a protocol for each patient, suggest things that need to be checked (e.g. laboratory values, kidney function) and highlight problems that have been found in a patient’s medication treatment. If all the comments offered by the physicians are combined, this provides a close description of what a medication review is and what the pharmacist generally does on the ward.

However, most physicians were unclear about the structure of the service and how it operates. For example, they did not know how to contact the pharmacist, when and how often the pharmacist visits the ward or if the pharmacist is still coming to the ward. This was more frequent amongst physicians that rotate between wards.

#### Suggestions to improve the service

The majority of physicians provided suggestions on how to improve and expand the clinical pharmacy service. These included: being able to call the pharmacist during working hours, education about drugs and pharmacological treatments, increasing the frequency of ward visits by the pharmacist, presentations on findings e.g. things physicians often miss, DRPs. The majority mentioned that they want the service to continue, and some physicians said that they want the service to grow and be implemented on other wards.

*“I believe that it is really good when [the pharmacist] comes, and that [the pharmacist] is a good asset and I hope that it will at least continue to be like this. That [the pharmacist] will come, that [the pharmacist] will be able to come more frequently.” (P3)*

Information and cooperation were described as key elements. As one physician explained, they *“… find a good form for it. That is, the physicians, and all staff, need to know that we now have a pharmacist here. So that they know it. So that I don’t start to look into things first, and then suddenly there is another person there who has a lot of good information. The cooperation must be formalized so that you know that on certain days there is this possibility, or at this time or that time.” (P6).*

### The clinical pharmacist

#### The value of the pharmacist in hospital wards

All physicians commented that it is very positive to have a pharmacist on the ward. As described by this physician: *“I believe that they [the pharmacists] have been an asset on the ward in an area that we, and by ‘we’ I mean physicians, often don’t have satisfactory knowledge in, i.e. drugs, interactions and such things. So I believe that they have contributed in a good way to the goal.” (P3).*

Participants described clinical pharmacists as “*helpful*” and “*supportive*”, an “*asset”* and *“a resource*”, but also as “*an expert that gives advice”, “a collaboration partner”, “a colleague*” and *“a colleague that has a special interest*”. Some of them also mentioned that pharmacists in general are underused in terms of the qualifications they possess. As illustrated by this physician’s comment: “*I believe that pharmacists in general are underused in Sweden. They study at university for ages and then sell medications at the [community] pharmacy.*” *(P4).*

The majority of the participants also mentioned that they did not know how much knowledge a pharmacist actually has until they started interacting with them. As expected, this differed depending on the physician’s experience with pharmacist-provided services. Before the service was implemented in these wards the majority of the participants considered the pharmacist’s main role is to work in a community pharmacy. As one physician described: *“Before I have always looked upon the pharmacist as someone working in a [community] pharmacy.” (P3).*

Most participants reported that they have interacted with pharmacists only during ward rounds or when community pharmacists have called them. In Sweden, community pharmacists contact the prescribing physician if questions about the prescribed medications arise. Some of the older physicians reported that besides the hospital wards they have interacted with pharmacists in drug and therapeutic committees (DTCs) or when using the service provided by the drug information centre at Västerbotten County Council (ELINOR). Physicians who had interacted with pharmacists in DTCs or ELINOR before the service was implemented in the hospital had more understanding of what knowledge pharmacists possess. This can be seen in the account of this physician: “*I have always had a vague idea about what a pharmacist does, until I participated in a drug and therapeutic committee. (…) After that I got a better understanding of what a pharmacist does. (…) They have much longer and more in-depth training than what I had believed.” (P6).*

#### The clinical pharmacist as the drug expert

Physicians often brought up drug knowledge during the interviews, both their own knowledge and that of the pharmacist. All the physicians described pharmacists as *“drug experts*” and repeatedly mentioned the pharmacists’ pharmaceutical knowledge. In particular, drug interactions and problems that emerge as a result of these were mentioned by most of the participants. Some of the younger participants mentioned that they themselves lack pharmaceutical knowledge or do not have as much knowledge as they would want. In contrast, older participants described how pharmacists complemented their own knowledge about drugs. As one physician said, *“Well [the pharmacist] has considerably deeper knowledge than I do about specific pharmacology, so to speak. And especially about interactions and such things that I’ve certainly read about some time ago but have forgotten.” (P7).*

#### Professional relationships

During the interviews the majority of physicians mentioned that they value the opportunity to interact with different health care professionals as they feel that each one can contribute with their expertise to patient care.

*“Like on the rounds, that you sit down with a physician, and a nurse, and an assistant nurse and the pharmacist. And everyone contributes with their expertise. The assistant nurse with nursing care and the physician with a little medical knowledge, and the pharmacist who has pharmaceutical knowledge. So all the pieces in the puzzle are there.” (P3)*

*“I believe it is splendid [to have different professions present during ward rounds]. Yes, we have different approach angles to the patients’ problems. So that other things can crop up that you have not really thought of.” (P7)*

When talking about collaboration, some of the physicians mentioned personal traits such as: “*you have to be humble*”, “*open to other individuals’ perspectives so that you can get along when you have different opinions*”, “*you cannot be a dictator*” and “*cannot take suggestions as criticism*”. According to these physicians, these characteristics are the basis of good teamwork.

*“You can’t take this as some sort of criticism. Instead, this is a really good opportunity to go through the medication lists with somebody who knows this on her five fingers.” (P5)*

Most physicians’ described that collaboration is valued but at the same time they described how ultimately they make the final decisions on how to treat patients, or as described by this physician: “*I have the final say*”. Physicians also commented on how they are used to managing things, making evaluations, calculating risks and determining the best way to treat patients. Most of the physicians see the pharmacist as part of the team. However, a couple of participants mentioned that if continuity improved (i.e. the pharmacist was available every day) they would be part of the team. A couple of participants also mentioned that continuity is important “so you learn to know each other”.

#### Professional barriers

All of the physicians pointed out that they themselves do not have any barriers to working with a pharmacist. However, there was a difference between how younger and older physicians describe this. All but one of the younger physicians mentioned that some of their senior colleagues might have difficulties working with pharmacists. One participant mentioned that this is a new way of collaborating and *“new things are not always viewed as positive”*. Another participant reported that they have noticed tensions between their colleagues and the pharmacists based on comments some physicians sometimes make. Some of the young participants also mentioned that they have not experienced tensions but believe that they could exist.

*“Some physicians appreciate this too, and some physicians believe that [the pharmacist] maybe is a little too skilled as [the pharmacist] checks everything to do with drugs and interactions and they want to override what [the pharmacist] says sometimes.” (P1)*

Older physicians however described that: *“I have no barriers”, “I don’t think that anybody has experienced that [barriers]”, “barriers exist between all professional roles”, “it is all about the person involved” and “it all depends on how things are put forth”*.

Some of the participants mentioned that personal traits and the “having the right approach” are important for pharmacists that work in hospital wards. According to these participants pharmacists must not be too demanding and not comment on everything they find on a patient. One participant noted that “physicians are people of habit”. Some physicians described how change could be hard, especially when challenging clinical autonomy. One physician reported: *“They [physicians] don’t want to be told what to do; you want to decide for yourself and some have more difficulties taking advice than others.” (P1).*

Even though all physicians claimed not to have barriers, some of their comments convey a somewhat different view. One physician said: “*Only physicians have knowledge of how to treat patients*”, only physicians know “*what works in real life*”*.* Some also mentioned that pharmacists only have “*theoretical knowledge*”. One physician commented: *“As a physician I have much more, and also nurses and assistant nurses, we all have more experience of working with a whole patient, with a human. While I can feel that the pharmacist’s point of departure is a little more of a theoretical one.” (P6).*

## Discussion

The aim of this study was to explore physicians’ perceptions regarding CPSs performed at hospitals operated by Västerbotten County Council. In this study, physicians were positive and supportive of the service and their collaboration with the pharmacist. Pharmacists were seen by physicians as drug experts and their recommendations were perceived as clinically relevant. All physicians wanted the service to continue and grow.

Physicians described benefits to themselves, for example how having a pharmacist in the ward allows them to perform other tasks, “learn” and improve their pharmacological knowledge. Consistent with the results of previous studies, physicians appeared to be satisfied with the service, were able to articulate the benefits for the patients and were likely to follow the advice provided by the pharmacist [22–24].

One interesting finding is that despite physicians’ positive attitude towards the service and clinical pharmacists, some noted they would like to perform the medication review themselves but the lack of time prevented them from doing it. Some also voiced strong views about clinical autonomy, being the final decision-maker and knowing what is best for their patients. This is in line with previous research where the role of the pharmacist has been described as being subordinate to that of the physician [25]. Over the years the role of the pharmacist has changed from traditional dispensing roles to more patient centred services [25]. Physicians maybe unaware of the professional skills of clinical pharmacists. When the clinical pharmacist and the physician start to interact and the pharmacist is able to demonstrate his/her competence, physicians may see the potential of clinical pharmacists and their role in the health care team. For example, a study performed by Zillich et al., showed that trustworthiness was positively associated with physician/pharmacist collaboration, and mentioned that after trust is developed, physicians are more willing to actively search for pharmacists’ advice [26].

McPherson et al. [27] noted that vocational pride, old habits, difficulties adapting to new things and feelings of *“I know how to perform my job”* and being threatened are barriers that hinder interprofessional collaboration (IPC). In a study about IPC between nurses, physicians’ and pharmacists, Ebert et al. [28] concluded that there is limited understanding of other health care professionals’ roles. As described by Chisholm-Burns et al. [9], working in multiprofessional health care teams involves a negotiated agreement between professionals who value the contributions and expertise that various health care professionals bring to patient care. One way to overcome these barriers is interprofessional education (IPE). An essential principle of IPE is that if students from different health professions learn together they will be better prepared for IPC via mutual understanding, communication and trust [29, 30]. Having a common goal is another thing that could also promote IPC.

Most physicians have little knowledge and awareness about how the service is delivered (e.g. scheduling, frequency), and saw this as an area for further improvement. Based on consultation with the head of the ward/department, clinical pharmacy has been tailored to each ward’s routines and needs. For example, some wards have set days and times while others do not. On wards that do not have set days and times the pharmacist decides when to attend ward rounds. Another reason for tailoring the clinical pharmacy is limited resources, as there is a limited number of clinical pharmacists employed. Awareness and resources may be a barrier to the expansion of clinical pharmacy services in Northern Sweden. Frequent changes in personnel (ward turnover and new physicians) can also create difficulties in terms of continuity, awareness and building interprofessional relationships [22]. It is interesting to note that the suggestions to improve the service provided by physicians (i.e. being able to call the pharmacist, presentation of statistics) are services that are currently being provided, highlighting once more the importance of increasing awareness.

### Implications for practice

To date, few wards operated by Västerbotten County Council have implemented CPSs. However, since this study was completed the service has been implemented in more wards and hospitals in the region. Still, the situation in Northern Sweden is similar to other countries [31, 32], not only in Europe but also elsewhere [33–35]. Physicians in this study described pharmacists as drug experts with pharmaceutical knowledge. Against this background, including clinical pharmacists in the health care team might optimize drug therapy and ensure patient safety, which could reduce DRPs and health care costs [15].

Physicians’ attitudes toward CPSs and collaborating with clinical pharmacists may facilitate or hinder the implementation and expansion of the services and the role of clinical pharmacist on hospital wards. For CPSs to be successfully implemented, to be sustainable and to grow, the roles, abilities and responsibilities of the clinical pharmacist need to be clearly described and there need to be opportunities to support collaborative relationships not only with physicians but with other health care practitioners. It is also important that decision-makers support the implementation of CPSs.

### Strengths and limitations

The results of this study need to be considered in the light of the limitations of the research. All of the interviewed physicians were positive and had similar views. Perhaps those who chose to participate were more likely to have positive views compared to those who decided not to participate. Furthermore, interviews were conducted by a Master’s in Pharmacy student and hence participants may have provided overall positive views. Participants might have felt uncomfortable expressing views that could be perceived as something negative about pharmacists. However, participants spoke freely during the interviews and the researcher perceived no implications of participants feeling uncomfortable. Also, the coders were all pharmacists, which might have contributed to a more positive interpretation of the results.

Interviews were conducted in Swedish and then reported in English. Considering that the purpose of the study was to convey views expressed by the participants, this could be difficult when quotes that are supposed to support the findings have to be translated as it is easy for their meaning to be “lost in translation” [36]. However, as previously mentioned, quotes were also back translated into Swedish. Culture and the way opinions are described may also have played a role. “Lagom” is a Swedish word meaning “just the right amount”. This is particularly reflected in the participants’ responses (not too negative, not too positive, just the right amount).

Even though the number of interviews conducted in this study was small, data saturation was reached. It is also important to highlight that the sample size also reflects the restraints of the small size of the current service. Currently, the service is only provided in some wards on certain days and by two clinical pharmacists. The number of physicians that have contact with pharmacists more than occasionally is limited. As described by Fusch and Ness [19] data saturation is not about the numbers but about the depth of the data. A small sample size may be more useful in examining a situation, while a large sample would be inconsequential. To date, most studies have focused on measuring health care providers’ satisfaction with CPSs via surveys [23, 33–35]. To our knowledge this is one of the few interview studies in this area conducted in Sweden.

## Conclusion

This study provides an in-depth exploration of physicians’ views and experiences of the service, provides insight into how physicians perceive the service and the pharmacists’ role and what they perceive to be the impact on patient outcomes. Since CPSs might be of special importance in Northern Sweden, the findings that all physicians were positive regarding CPSs and were satisfied with the collaboration with the clinical pharmacists, are important to put forward in order to further implement and expand CPSs.

## Additional file

Additional file 1: Interview schedule. The interview schedule used, translated from Swedish to English. (PDF 54 kb)

## Abbreviations

ADE: Adverse drug event
ADR: Adverse drug reaction
CPS: Clinical pharmacy service
DRP: Drug related problem
DTC: Drug and therapeutic committees
ELINOR: Drug information centre at Västerbotten County Council
IPC: Interprofessional collaboration
IPE: Interprofessional education
PPH: Potentially preventable hospitalizations
