# Supplementary material for: Physicians’ perspectives on clinical pharmacy services in Northern Sweden: a qualitative study
Source: BMC Health Serv Res. 2018 Jan 24;18:35. doi: 10.1186/s12913-018-2841-3 (PMC5781320; doi:10.1186/s12913-018-2841-3)
Supplement: Additional file 1: — Interview schedule. The interview schedule used, translated from Swedish to English. (PDF 54 kb) [file 12913_2018_2841_MOESM1_ESM.pdf]

Additional file 1. Interview schedule [Translated from Swedish]

Pharmacists on the ward

1) Can you tell me about your experience of working with clinical pharmacists on the ward?

Prompts

- When do you meet the pharmacist on the ward?
- Are rounds carried out on your ward? How are they performed? Who participates? Does the pharmacist take part in the rounds?
- What is your opinion concerning the different professionals participating in the rounds?
- What is your general view of clinical pharmacists?

2) Can you describe what the pharmacist does on your ward?

Prompts

- Can you describe how the pharmacist works? What do you think about this?
- Is there something in particular that the pharmacist does that works well? Something that does not work so well?
- Does the pharmacist do other things on your ward?

3) What is your view of the recommendations given by the pharmacist??

Prompts

- Are the recommendations adequate? Can you give me some examples?
- What is your opinion of the clinical relevance of the recommendations?

4) Are there any benefits to having a pharmacist on the ward?[Yes/No] Can you give some examples?

Prompts

- What are the advantages? Any disadvantages?
- Can the pharmacist contribute to the work of the patient care team, and if so can you please give examples?

Outcomes

5) What are your thoughts on having a ward-based clinical pharmacist?

Prompts

- Are there any advantages? Are there any disadvantages? For the patient?
- In what way can the pharmacist's role impact on patient-safety? Can you please give some examples?

I have two concluding questions about the future of ward-based clinical pharmacists.

6) How do you see the future of pharmacists working clinically in hospital wards?

- Barriers?
- Enablers?

7) Do you have any suggestions?
